# 

**Published:** 1910-04-09

**Authors:** 


### Cough and the Uvula.

In the sucking infant the uvula normally lies against the posterior aspect of the epiglottis, thus providing a median channel for respiration, while the flow of milk in sucking proceeds on either side. So that the assertions made regarding the causation of cough by the contact of the uvula with the epiglottis do not rest on a very secure foundation.

### Administration of Iron.

Research has done much to dissipate the notion that ferruginous preparations are beneficial only by being directly assimilated. More iron is prescribed in a day, if not in a dose, than would be normally contained in the entire body of a healthy person. It has been shown that most, if not all, the metal is recoverable in the fæces, yet it is without doubt beneficial in chlorosis, indicating that, apart from absorption, the presence of iron exerts some beneficial action in the digestive tract, partly by counteracting other chemical bodies by producing insoluble combinations. This may be the case with the sulphides of ammonium and hydrogen, since the iron is disposed of as sulphide, and these compounds are capable of entering into undesirable combinations with hæmoglobin.—*Dr. Essex Wynter.*

### The Midwives Act.

On the first day of this month those provisions of the Midwives Act which have not hitherto been operative came into force, and henceforward it is illegal for anyone not on the roll kept by the Central Midwives Board to practise, habitually and for gain, the office of midwife. At one time, and not so very long ago, apprehensions were freely expressed that a serious shortage of midwives would ensue, and that the poor would therefore be hampered and oppressed by the working of these clauses of the Act. It was thought that in many districts women might have to suffer the pangs of labour unattended by any except amateur helpers; and it was commonly predicted that the provisions of the Act would prove inoperable for this reason. From returns issued by the several counties it would seem that in England and Wales there are but fifteen counties in which even temporary difficulty is expected. It is perhaps too much to hope that for the next year or so there will be absolutely no instances of hardship owing to the provisions of the Act; but it is by now quite certain that as a whole the community has already profited enormously from those provisions, and probably will in the future benefit even more. It may also be hoped fairly confidently that cases of distress will be far fewer than was at one time thought probable, and that local authorities all over the country will do their very utmost to prevent them as far as possible.

### THE GRADUATES' MEDICAL SCHOOLS.

DIARY FOR THE WEEK, APRIL 11 to APRIL 16.

### CENTRAL LONDON THROAT AND EAR HOSPITAL, Gray's Inn Road, W.C.

At 3.45 p.m.—**April 15**, Dr. Abercrombie, **Clinical Cases.**

### ROYAL SOCIETY OF MEDICINE, 15 Cavendish Square, W. (Temporary address during building.)

At 7.45 p.m.—**April 14, Obstetrical and Gynæcological Section:**

*Specimens.*—Dr. A. E. Giles : Large fibroid of cervix.

*Paper.*—Dr. Henry Jellett : "The place of Cæsarean section in the treatment of placenta prævia."

8.30 p.m.—**April 15, Electro-Therapeutical Section:**

*Paper.*—Dr. Howard Pirie : "The disappearance of enlarged glands in lymphadenoma under treatment by *x*-rays."

*Discussion.*—"The relative value of the various types of high tension transformers used for the production of *x*-rays," opened by Dr. Reginald Morton.

*Demonstrations.*—Dr. Howard Pirie : A pocket stereoscope for use with *x*-ray negatives. Mr. A. J. H. Iles : A new simple stereoscope.

### SOME MANCHESTER SPECIALITIES.

(Medical Requisites Co., 54 Deansgate, Manchester.)

This firm supplies a number of interesting specialities. To one of these—the telescopic splint—we have alluded elsewhere. The Facile Ideal Operating Table and Couch is a new and improved form of surgical operating and examining chair and couch combined. Its mechanism, while admitting of all possible necessary movements, is simple, and it is so strongly made that it is securely stable in any position. It can be manipulated with the greatest ease, and is provided with the usual extras in the shape of foot rests, movable arm and head rests and straps. Special abdominal rests are provided by means of which it is comparatively simple to secure variations of the Trendelenburg position, or to assist the surgeon in gall, bladder, or kidney operations. The firm supplies an illustrated descriptive booklet. The Litra self-adjustable bedrest and the Beech massage machine are also supplied by this firm. The first is one of the simplest and therefore most desirable bed-rests in the market. We have thoroughly tested a specimen supplied by this firm, and can, therefore, speak from experience of its merits. It is a most durable article, and not the least interesting point about it is that it can be easily sterilised. The wire mesh rests appear to us to be the most suitable for institutional use, but the steel lath models are stronger, though considerably heavier. An ideal Litra would, we imagine, be of aluminium bronze, with " flat" netting, which is much more easy to clean. The Beech massage machine is a vibratory instrument for instrumental massage, and is recommended for the massaging of the face and head. We have no practical experience of this machine, but it appears to be simple in construction and easily worked by either direct or alternating current motor. For institutional use such a machine is scarcely necessary, but those who wish to acquire a vibratory massage instrument would do well to inspect the Beech model before fixing upon any make.

### APPOINTMENTS.

Dr. H. F. Heath, Director of Special Inquiries and Reports, has been appointed to the post of Principal Assistant-Secretary of the Universities Branch of the Board in combination with his present post.

The Board of Management of the Manchester Royal Infirmary has made the following appointments:—House physicians: R. C. Harkness, M.B., Ch. B. (Edin.), J. Ramsbottom, M.B., Ch.B. (Vict.); Medical officer, central branch: Robert Ollerenshaw, M.D. (Vict.), F.R.C.S. (Eng.).

**For Hospitals and Institutions.**

| | |
|---|---|
| Analysis Ledger. 3 vols. (Uniform with the new Index of Classification adopted by the King's Hospital Fund) | £1 12s. 6d. |
| Cash Book | 12s. 9d. |
| Cash Analysis and Receipt Book | 16s. 6d. |
| Secretary's Petty Cash Book | 13s. 0d. |
| List or Register of Annual Subscribers | £1 0s. 0d. |
| Linen Register | 7s. 6d. |

And many other Ledgers necessary in the office of large and small institutions.

Of all booksellers or of The Scientific Press, Limited, 28 and 29 Southampton Street, Strand, London, W.C.

### BOOKS RECEIVED.

Adolphe Francis, Ltd.

"The British and Foreign Guide to the Chemical, Drug, and Oil and Colour Trades."

J. and A. Churchill.

"Lessons on Elementary Hygiene and Sanitation, with Special Reference to the Tropics." By W. T. Prout, C.M.G., M.B., C.M.

George Bell and Sons.

"American Meat." By Albert Leffingwell, M.D.

William Rider and Son, Ltd.

"Nature's Help to Happiness." By J. Warren Achorn, M.D.

"Nervousness." By A. T. Schofield, M.D.

Henry Frowde, Hodder and Stoughton.

"The Hospital Service Book." By C. T. Baxter, M.A.

"The Expectation of Life of the Consumptive after Sanatorium Treatment." By N. D. Bardswell, M.D., etc.

"Practical Pathology." By G. Sims Woodhead, M.A., etc.

"Consumption: its Prevention and Home Treatment." By H. H. Thomson, M.D.

"Operations of General Practice." By E. M. Corner, M.A., F.R.C.S., and H. Irvine Pinches, M.A., M.B., M.R.C.S., L.R.C.P.

W. H. and L. Collingridge.

"City of London Year-book," 1910.

Headley Bros.

"The Prayer Quest." By W. Winslow Hall.

### Jottings.

The annual meeting of the Eye and Ear Hospital, Tunbridge Wells, which had been postponed for a month owing to the death of Colonel Holland, C.B., who had been for many years president of the governors, has now taken place.

The president of the General Hospital, Cheltenham, Colonel Croker King, remarked at the annual meeting that he had approached the police authorities and the watch committee in connection with the admission of cases of attempted suicide. He thought that the police authorities should pay something towards the wages—5s. by day and 5s. by night—of the man who looked after them.

The cot which public generosity has endowed, in memory of the late Canon Fleming, has now been dedicated in the Victoria (Children's) Ward of the York County Hospital. The occasion was a public ceremony, given peculiar meaning because Canon Fleming, as Mr. J. Melrose reminded those present, had contributed by his famous readings to the hospital in his lifetime.

Princess Christian has given her patronage to the three dramatic performances organised by Florence Lady Clarke-Jervoise, which will be given at the New Royalty Theatre on the evenings of April 11, 12, and 13. A new one-act play by Lady Clarke-Jervoise entitled *Shub'rat* ("Night of Record") and *Lady Epping's Law Suit* will be performed on each occasion. The first performance will be in aid of the Rev. the Hon. James Adderley's work in East Birmingham, and on the following day the receipts will go to the fund which Lady Clarke-Jervoise is collecting to endow a cot in the East London Hospital for Children at Shadwell, E., while on the third evening "Our Dumb Friends League" Animals' Hospital is to benefit.

### Gifts and Bequests.

Mrs. Mary Sturdy, of Lindfield, Sussex, has left £300 to the Elliot Cottage Hospital, Haywards Heath.

Miss Mary Ann Blatherwick, of Haywards Heath, has left £300 to the Hospital for Sick Children, Great Ormond Street.

The Treasurers of the Middlesex Hospital have received a donation of thirty guineas from the "D" Division of the Metropolitan Police.

The Leicester Infirmary and House for Recovery from Fever have received the sum of £100, bequeathed by the will of Mr. Wates, of Bromley, Kent, to these institutions.

Miss Emily Miller, of Russell Square, aged seventy-one, who left estate of the gross value of £17,620, left from the residue of her estate, subject to the payment of duties, one moiety to the North London Consumption Hospital.

The late Miss Oliver, who died in a Paris slum, and who, when about ten years old, attended the opening of the Royal Berkshire Hospital, has left to that institution a sum of money estimated at £300, and to Guy's Hospital £2,800.

Mr. Arthur Walwyn Burdett, of Knowle, Warwick, has left (on the death of his wife and after certain bequests) one-half of the balance of his estate upon trust (1) to endow a bed in the Birmingham General Hospital; (2) to endow a bed in the Queen's Hospital, Birmingham; (3) balance to the Medical Benevolent Society, with offices at 93 Cornwall Street, Birmingham, upon trust to apply the income for the benefit of widows and daughters of deceased medical men who have practised in the counties of Warwick and Leicester, this fund to be called the "Henry Francis Burdett Memorial Fund."

### THE QUESTION BOX.

**SPECIAL NOTICE.**—Questions of interest affecting the honorary medical staff and hospital administration in all departments are invited. When any point arises we hope our readers will formulate it in a question and so co-operate to make this column of real value and interest. In this way the experience of the best-managed hospitals should be made helpful to the whole hospital world. We shall welcome the contribution of both questions and answers.

N.B.—The publication of an answer in this column does not necessarily preclude the publication of subsequent answers to the same question, for a question may involve points which require to be threshed out and fully discussed. Answers, if published, will be paid for at scale rates.

### THE PURCHASING OF PROVISIONS.

Question: Is it in the best interests of an institution for all provisions, etc., to be purchased by and to be under the direct control of the Matron's or the Secretary's department?

### A Provincial View.

By the Secretary and House Governor of a Large Provincial Hospital.

It will surely occur to your readers that the interests of most institutions will be best served by such an arrangement as will bring both the administration and management into harmonious working and co-operation on this important point.

The issue of provisions is, I suggest, a matter in which the matron or those acting under her—*e.g.*, her assistant or the housekeeper—is probably much more likely to take kindly to and to perform satisfactorily than the Secretary, and may therefore be placed under her care.

Where, however, there are complaints as to the quality of provisions or stores, or where the supply is such as to cause inconvenience, these are matters which it is desirable should be dealt with by the Secretary, who will advise as to remedial measures, and act as intermediary between the institution and the contractor, with a view to assuring smooth working.

Almost without exception provisions are purchased by contract—usually for six or twelve months. These contracts are drawn up by the Secretary and issued from his office, and when returned are tabulated by him and submitted to the committee, with the samples. It is essential that the tabulation should contain a column as to whether the past supplies of the firm in question have been satisfactory and delivery prompt and convenient; if not, defaults should be stated. This should be made in consultation with the matron or the officer issuing supplies, for it is obvious that if the institution is to work smoothly the officers in question must be protected from the contractor who has a bad reputation.

Contracts having been examined and accepted by the committee, according to varying customs at various institutions, copies should be made for the matron or the stores officer, who will order her supplies thereon. Counterpart orders should be sent to be filled up and returned by the contractor with the supplies; if correct as to quantity and quality, the counterparts will be initialled by the receiving officer and referred to the Secretary for checking against the tenders and subsequent payment.

These remarks apply to the purchase and issue of provisions only. Doubtless "Etcetera" in the question is not intended to have a general application.

### MEDICAL AND SURGICAL STATISTICS IN HOSPITAL REPORTS.

Question: Has any hospital at present made an attempt to remodel these statistics and make them represent something definite and prove of scientific value, which each governor and the public generally may grasp and understand?

### A Resident Medical Officer's View.

The London Hospital has now a statistical department, of which the director is Mr. Major Greenwood, junr., M.R.C.S., L.R.C.P., a pupil of Professor Karl Pearson. In general, however, figures relating to number of attendances and of operations are collected by house physicians and house surgeons, and presented in the annual report almost undigested; failing the appointment of a special officer to deal with the matter, this rather slovenly way of doing things is likely to continue. One obvious opening for profitable research is the occupational incidence of disease, and many interesting facts, some of them of great possible local importance, might be brought to light. But, cynical as the observation may appear, nothing much is likely to be done on these lines, at any rate for some time, because the British clinician's idea of research connotes the use of the microscope or of the apparatus of the physical laboratory—hardly anything but that; and what interests him he is more likely to record in his own case-book than in his hospital report. Indeed, under present conditions he can hardly be expected to be altruistic enough to act otherwise. The tone of the above question, suggesting, namely, that it is a pity more use is not made in hospitals of collective investigation, seems to me fully justified.

